# Ultrafast Spin Dynamics in 2D Fully Compensated Ferrimagnets: A Time-Dependent Ab Initio Study

**DOI:** 10.1021/acs.jpclett.5c02874

**Published:** 2025-10-17

**Authors:** Shuo Li, Ran Wang, Thomas Frauenheim, Sandong Guo, Zhaobo Zhou, Junjie He

**Affiliations:** † Institute for Advanced Study, 74707 Chengdu University, Chengdu 610106, China; ‡ School of Science, Constructor University, Bremen 28759, Germany; § School of Electronic Engineering, Xi’an University of Posts and Telecommunications, Xi’an 710121, China; ∥ Department of Physical and Macromolecular Chemistry, Faculty of Science, 37740 Charles University, Prague 12843, Czech Republic

## Abstract

Fully compensated ferrimagnets (CFiMs) represent a novel class of magnetic materials that combine zero net magnetization with strong spin polarization, offering considerable potential for spintronic applications. Here, we employ real-time time-dependent density functional theory (rt-TDDFT) to investigate ultrafast laser-induced spin dynamics in a two-dimensional (2D) Janus NiICl bilayer. The broken inversion symmetry gives rise to an asymmetric interlayer demagnetization process, leading to a transient net magnetization in this system within 50 fs. This phenomenon is attributed to the asymmetric charge accumulation and interlayered optically induced spin transfer (OISTR) between the two Ni magnetic sublattices, facilitated by the intrinsic structural and electronic asymmetry of the Janus configurations. The asymmetric interlayer interaction effectively leads to a transient ferrimagnetic state. Our work reveals the microscopic mechanism of the ultrafast spin dynamics of 2D CFiMs induced by lasers in ultrafast spintronics.

## Graphical abstract

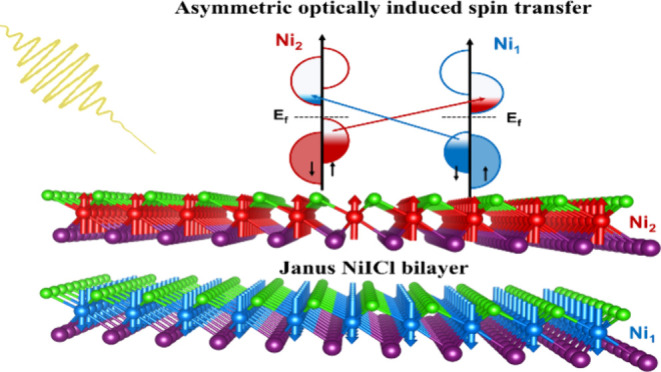

Magnetic materials^1^ are conventionally categorized into three distinct classes: ferromagnets (FMs), antiferromagnets (AFMs), and ferrimagnets (FiMs). FMs exhibit strong spin polarization and finite net magnetization, rendering them highly suitable for spintronic applications.^2,3^ However, significant stray fields frequently result in undesirable crosstalk and energy dissipation in high-density devices.^4^ Conversely, AFMs exhibit no net magnetization and exhibit considerable resistance to external magnetic fields.^5,6^ However, their absence of spin polarization limits their application in spin-dependent transport. Altermagnetism,^7–9^ a recently emergent phenomenon, represents a novel class of magnetism that combines zero net magnetization with strong spin-split band structures. These structures are enabled by specific crystal symmetries, thereby merging the advantages of both ferrimagnets and antiferromagnets.^10^ Concurrently, fully compensated ferrimagnets (CFiMs) have attracted a growing interest.^11^ In these cases, compensation arises from electron filling rather than symmetry, a phenomenon that is frequently observed in collinear magnetic semiconductors or half-metals.^12–14^ CFiMs maintain a sublattice configuration analogous to that of conventional ferrimagnets, thereby providing an alternative method for the integration of spin polarization with magnetic compensation. Despite their potential, the physical mechanisms and application prospects of CFiMs remain underexplored.

Laser-induced ultrafast magnetic phase transitions and spin dynamics represent a pivotal frontier in spintronics, with the potential to manipulate magnetic order on femtosecond time scales.^15^ This has the promise of dramatic improvements in speed and efficiency for information storage and processing.^16^ Conventional approaches to magnetic control, including external magnetic fields, electrical gating, and strain, are typically characterized by slow response times and high energy costs.^17–19^ In contrast, ultrafast optical excitation has been demonstrated to trigger rearrangements of magnetic order on femtosecond-to-attosecond scales, facilitating rapid switching and efficient spin control.^20–23^ A pivotal mechanism in these processes is the optically induced spin transfer (OISTR),^24^ wherein laser pulses promote spin-dependent charge transfer between atomic sites or layers, generating nonequilibrium spin populations that can manipulate magnetic states.^25–28^ Despite the fact that laser-driven dynamics in FMs and AFMs have been the focus of extensive study over the past few decades^29–32^ and that emerging research has begun to address altermagnetic systems, the ultrafast magnetization dynamics of fully compensated ferrimagnets (CFiMs) remain largely uncharted territory. A systematic investigation is imperative to elucidate the spin behavior in these systems and evaluate their potential for next-generation spintronic devices.

In this work, a novel approach is proposed to overcome the limitations of conventional magnetic materials by designing a two-dimensional (2D) CFiM based on the Janus NiICl bilayer.^11,33,34^ This structure inherently disrupts inversion symmetry due to its heterogeneous surface termination of I and Cl atoms, giving rise to asymmetric electronic environments and spin-polarized band structures. In contrast to symmetric antiferromagnetic systems, the NiICl bilayer displays distinct layer-differentiated demagnetization behavior upon exposure to laser excitation. The present study systematically investigates the ultrafast spin dynamics of the material and demonstrates that optical pulses induce a transient net magnetic moment through asymmetric interlayer spin transfer and site-selective charge accumulation. This work demonstrated the mechanisms driving laser-induced magnetization in the 2D CFiMs, with the role of structural asymmetry and nonequilibrium spin populations being highlighted.

The optimized structure of the NiICl bilayer with the Cl–Ni–I Janus configuration is shown in Figure 1a. To estimate the structural stability of the NiICl bilayer, we calculated the binding energies, which are defined as *E*_b_ = *E*_(NiICl bilayer)_ – 2*E*_(NiICl monolayer)_. Here, *E*_(NiICl bilayer)_ and *E*_(NiICl monolayer)_ are the total energy of the NiICl bilayer and the isolated NiICl monolayer, respectively. *E*_b_ is −0.10 eV per unit cell, thus reflecting weaker interlayer interactions. Owing to the antiferromagnetic coupling between the Ni_1_ and Ni_2_ layers, the heterostructure adopts a fully compensated ferrimagnetic (CFiM) state with zero net magnetization. The inherent Janus configuration of NiICl breaks the inversion symmetry, effectively decoupling the two magnetic sublattices by situating the I and Cl atoms in distinct local environments. Furthermore, the charge density difference of the NiICl bilayer, as shown in Figure S1, reveals a built-in vertical electric field induced by asymmetric interlayer van der Waals interactions. Figure 1b displays the band structure of the NiICl bilayer, which exhibits pronounced spin splitting. The band structures of the NiICl bilayer and the magnetic moments of the Ni atoms at different *U* values are shown in Figure S2. Additionally, the projected band structures in Figure 1c illustrate the contributions from Ni_1_ and Ni_2_ atoms, indicating that the conduction band minimum (CBM) and valence band maximum (VBM) in the spin-up channel are primarily derived from Ni_1_ and Ni_2_ atoms, respectively. The projected density of states (PDOS) of Ni, I, and Cl atoms, shown in Figures 1d–1f, further confirms spin splitting across all atomic species.

**Figure 1:**
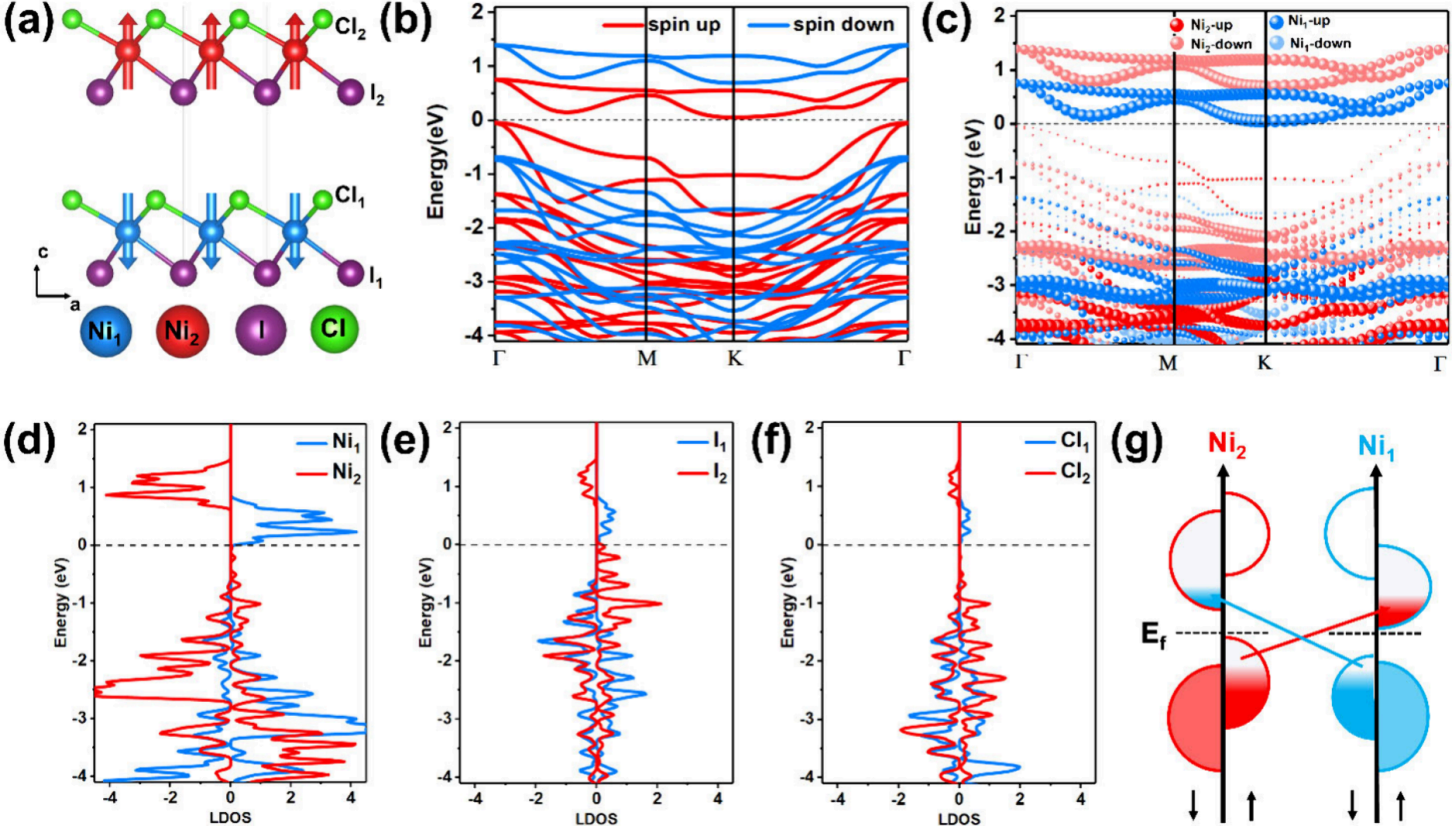
(a) Crystal structure of the NiICl bilayer with two AFM spin sublattices. Blue, red, purple, and green spheres represent Ni_1_, Ni_2_, I, and Cl atoms, respectively. Blue and red arrows represent the opposite Néel vectors of two Ni atoms in the unit cell. (b) Band structure of the NiICl bilayer without the spin–orbital coupling (SOC) effect. (c) Projected band structure of Ni_1_ and Ni_2_ atoms in the NiICl bilayer without SOC. (d, e, and f) The projected density of states of Ni, I, and Cl atoms. (g) Schematic of an asymmetric OISTR process in the NiICl bilayer. Red and blue arrows represent the spin transfer in two spin sublattices.

The unique electronic structure of the NiICl bilayer, characterized by broken inversion symmetry and strong spin splitting, makes it a compelling platform for investigating photoinduced ultrafast spin dynamics. In such systems, a mechanism known as the optically induced spin transfer (OISTR) effect facilitates the subfemtosecond transfer of spin-polarized electrons between atomic layers or sublattices, resulting in nonequilibrium spin populations.^24^ In conventional antiferromagnets, the magnetic layers are electronically equivalent, leading to a symmetrical OISTR response under laser excitation. In contrast, the NiICl bilayer exhibits pronounced layer-resolved asymmetry in both the electronic structure and magnetic environment. This asymmetry enables a differential OISTR response, where spin-polarized electrons undergo distinct excitation and transfer dynamics within each magnetic layer. Such an imbalance is expected to disrupt the compensation between spin sublattices at ultrafast time scales in 2D CFiMs.

To investigate the laser-induced spin dynamics in 2D CFiMs, we performed real-time time-dependent density functional theory (rt-TDDFT) simulations to compare the ultrafast spin responses of the NiICl bilayer. In both cases, we applied an ultrashort, linearly polarized laser pulse with a full width at half-maximum (fwhm) of ∼10 fs and a fluence of 12.29 mJ/cm^2^ (Figure 2a). To further elucidate the ultrafast demagnetization process and the emergence of laser-induced net magnetization in the NiICl bilayer, we present the time-dependent element-resolved spin dynamics in Figure 2. For clarity, the change in magnetic moment, defined as Δ*M* = *M*(*t*) – *M*(*t* = 0), is shown in Figures 2b–2d. Both Ni_1_ and Ni_2_ exhibit pronounced ultrafast demagnetization following laser excitation; however, their temporal spin dynamics differ significantly. This asymmetry in the magnitude and timing of spin loss between the two magnetic layers induces a transient imbalance, giving rise to a fluctuating net magnetic moment of approximately 0.03 μ_B_ within 50 fs. Moreover, the changes in spin polarization for I and Cl atoms are comparatively weaker; they also contribute to a slight fluctuating net moment. Consequently, the difference between Ni_1_ and Ni_2_ in the NiICl bilayer is due to the asymmetry of the interlayer exchange interactions, leading to a different spin-transfer mechanism, which is a key contributor to the emergence of the net magnetic moment in 2D CFiMs.

**Figure 2:**
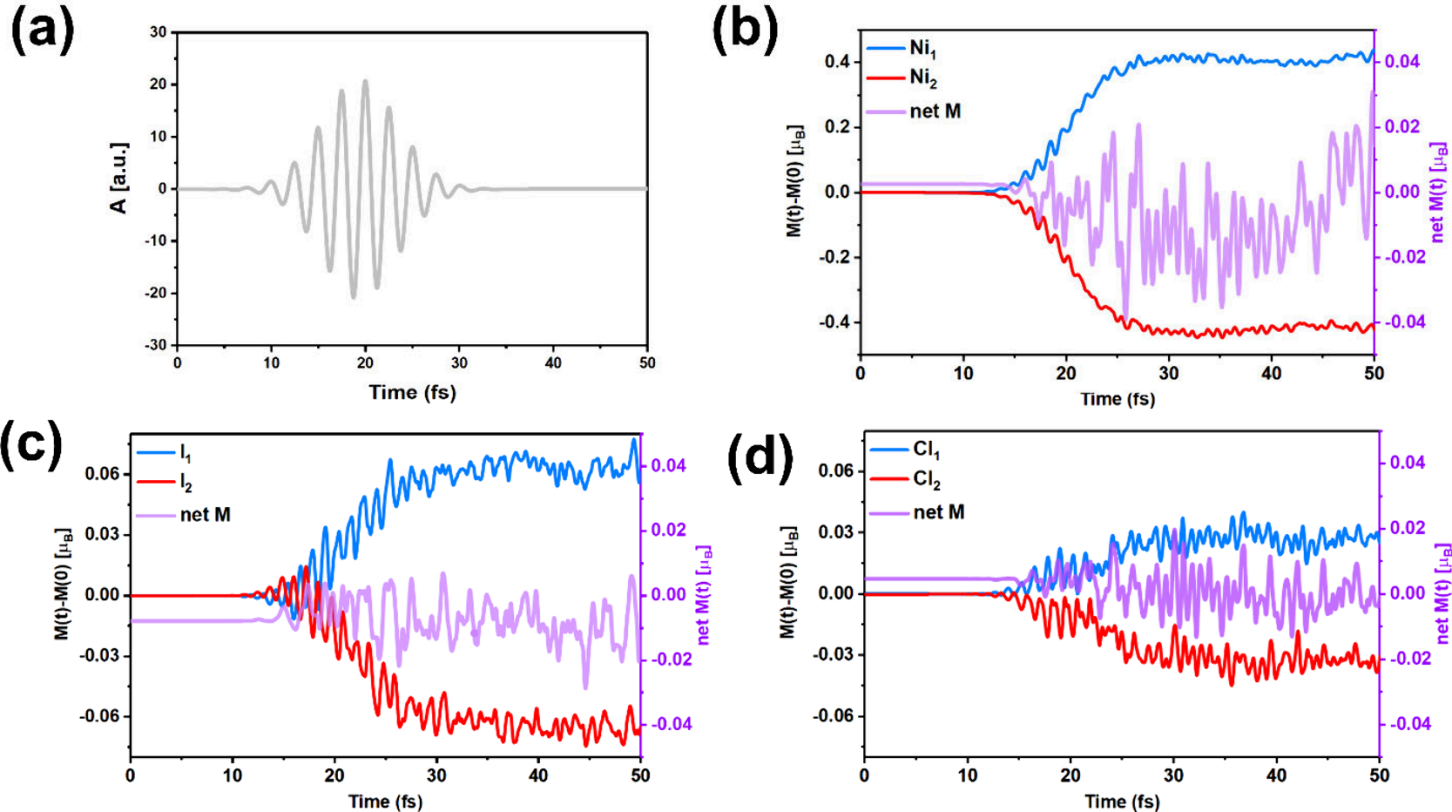
(a) Vector potential of the laser pulse (the frequency *f* = 1.63 eV, the full width at half-maximum fwhm = ∼10 fs, and the fluence *F* = 12.29 mJ/cm^2^). Normalized (b) Ni, (c) I, and (d) Cl atom-resolved spin moment as a function of time, respectively. The related net magnetic moment (net M) is shown in purple.

In order to further visualize this ultrafast nonequilibrium spin dynamics process in the NiICl bilayer, the time-dependent spin density dynamics were calculated (Figure 3). The results obtained clearly demonstrate the asymmetric demagnetization dynamics and spin transfer flow of the NiICl bilayer. The presence of two NiICl layers results in an analogous demagnetization. However, the asymmetric interlayer interaction between Cl_1_ and I_2_ in the NiICl bilayer results in the electronic asymmetry. This interfacial asymmetry facilitates unequal net spin transfer flow, emphasizing the significance of chemical and structural heterogeneity in controlling ultrafast magnetic responses.

**Figure 3:**
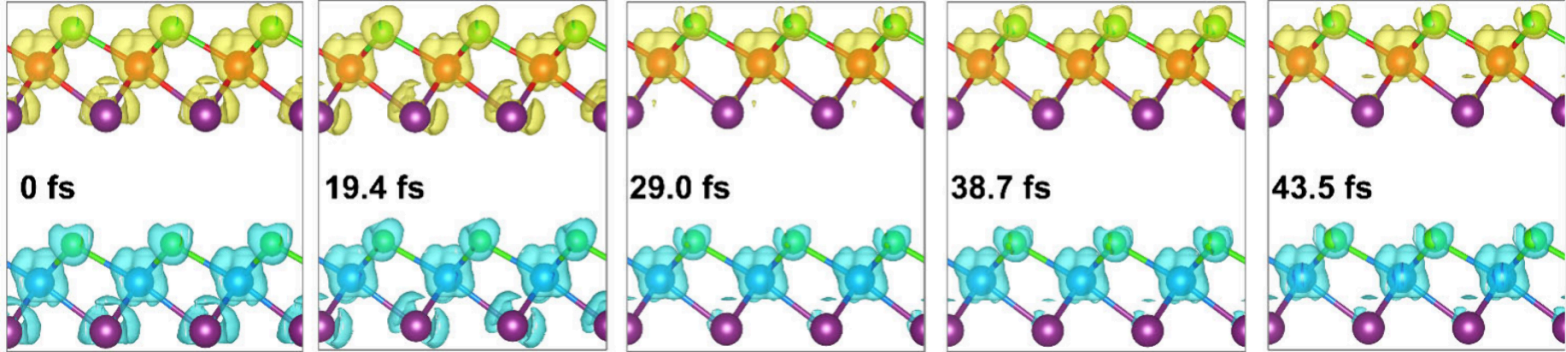
The time-dependent magnetization density of the NiICl bilayer. Yellow and blue domains indicate the spin-up and spin-down density, respectively. The isosurface is set to 0.005 *e*/Bohr.^3^

To further understand the role of the charge excitation in the spin dynamics, we calculated the change in time-resolved DOS (ΔDOS­(*t*)) for Ni_1_ and Ni_2_ atoms. This is defined as the difference between the DOS at time *t* = 43.5 fs and *t* = 0, as shown in Figure 4a. The results reveal a distinctly asymmetric charge accumulation process, wherein electrons preferentially occupy the conduction band minimum (CBM) associated with the Ni_1_ atom in the spin-up channel and the Ni_2_ atom in the spin-down channel. This site-selective and spin-selective population leads to an asymmetric demagnetization within the NiICl bilayer, ultimately resulting in the emergence of a net magnetic moment across the nickel sublattices. Consequently, the direction-dependent, spin-selective charge transfer underpins the observed asymmetric spin dynamics, which directly underlies the enhanced demagnetization rate. This mechanism is further corroborated by the detection of a spin-polarized asymmetric current flow.

**Figure 4:**
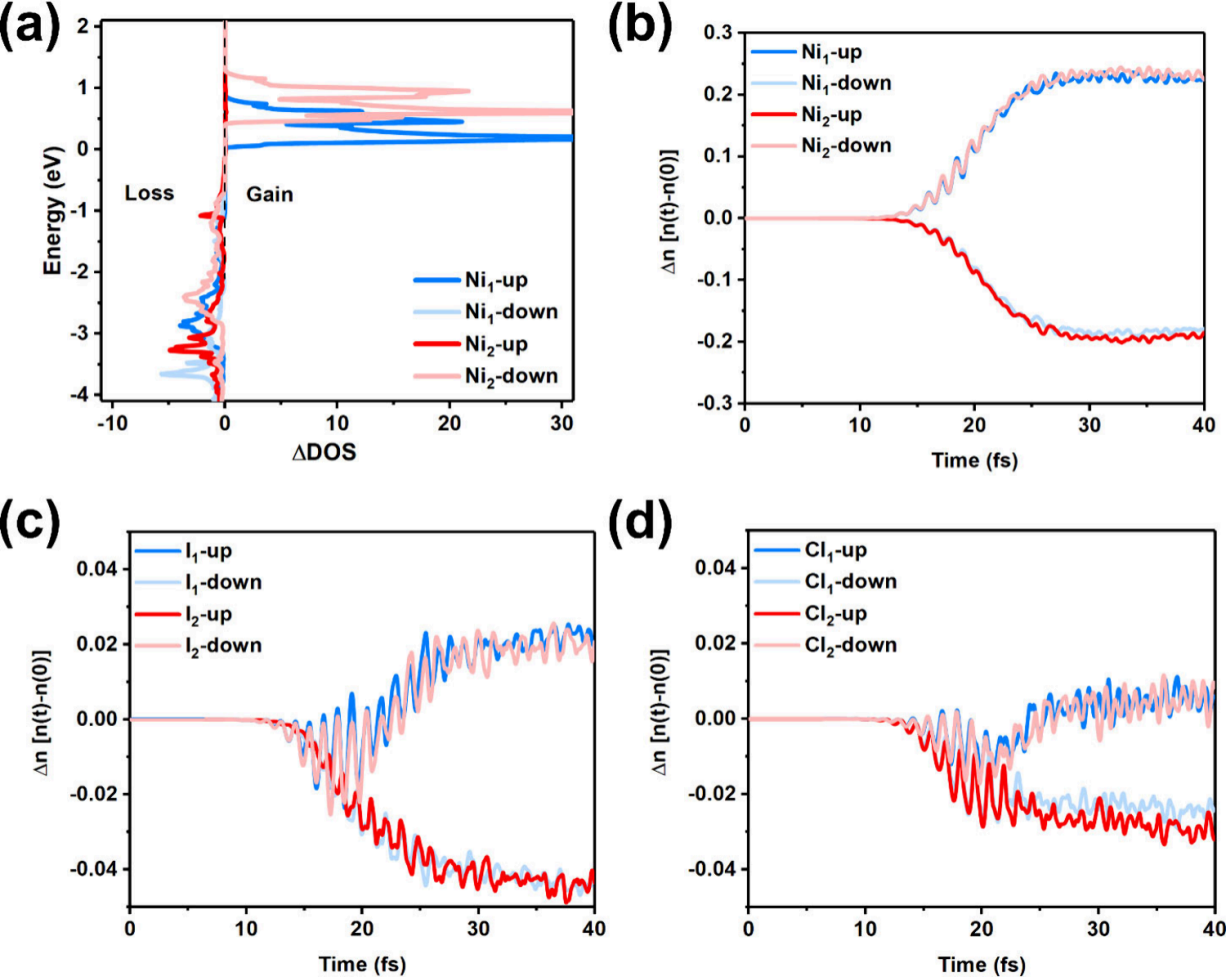
(a and b) Differences in the time-resolved occupation function ΔDOS­(*t*) at *t* = 43.5 fs. The negative value signifies a loss of electrons, and a positive value signifies a gain of electrons. (c and d) Change in the spin-resolved charges Δ*n* of two Ni atoms. The positive/negative value represents the increase/decrease of charges.

Furthermore, the time-dependent changes in spin-resolved charge Δ*n* of Ni atoms are shown in Figure 4b, respectively. The utilization of spin-resolved charge dynamics has been identified as a means of characterizing alterations in spin moment loss, which is expressed as follows

$$\Delta n_{↑}\left(t\right)=\left[\Delta n\left(t\right)+\Delta M\left(t\right)\right]/2$$

$$\Delta n_{↓}\left(t\right)=\left[\Delta n\left(t\right)-\Delta M\left(t\right)\right]/2$$

where Δ*n*_↑_(*t*) = *n*(*t*) – *n*(*t* = 0) represents the change in local charge compared to the initial charge. Δ*n*_↑_(*t*) and Δ*n*_↓_(*t*) denote the time-dependent changes in spin-up and spin-down charges, respectively. The change in spin moment can be defined as Δ*M*(*t*) = Δ*n*_↑_(*t*) – Δ*n*_↓_(*t*), where the higher the difference between Δ*n*_↑_(*t*) and Δ*n*_↓_(*t*) the more significant the loss in spin moment. The results demonstrate that Δ*n*_↑_(*t*) and Δ*n*_↓_(*t*) of Ni atoms exhibit an increase and decrease, suggesting the demagnetization process of Ni atoms in the NiICl bilayer. Moreover, the asymmetric Δ*n* of Ni_1_ and Ni_2_ is observed in Figure 2b. Moreover, the asymmetric Δ*n* of I and Cl atoms is also observed in Figure 2c and 2d, but it is smaller than that of Ni atoms. Consequently, the asymmetric redistribution of the spin-resolved charge between Ni_1_ and Ni_2_ further corroborates the asymmetric OISTR and generation of ferrimagnetic polarization in the NiICl bilayer.

While our simulations resolve the ultrafast spin dynamics within the first 50 fs, the influence of electron–phonon coupling on longer time scales remains an open question. Further investigations are necessary to determine whether the induced net magnetic moment can sustain its growth over extended periods, potentially culminating in a phase transition to the ferromagnetic order. Understanding the interplay between phonon-mediated excitations and spin relaxation in 2D CFiMs is crucial, as it may govern the stability and dynamical evolution of the nonequilibrium magnetic state.

Notably, the observed net magnetic moment in the NiICl bilayer remains small, which may limit its practical utility in device applications. To increase this magnitude, strategies such as applying external electric or strain fields to amplify the electronic asymmetry, optimizing the Janus structure to strengthen the interlayer spin polarization, or exploring alternative material systems with stronger SOC or magnetic interactions could be further explored. Increasing the net moment would improve the signal strength in spintronic applications and provide a broader platform for studying nonequilibrium magnetic phase transitions in 2D CFiMs.

In summary, this study employs rt-TDDFT to investigate ultrafast laser-induced spin dynamics in the Janus NiICl bilayer. Our simulations reveal that photoexcitation triggers pronounced layer-asymmetric demagnetization, breaking magnetic compensation and inducing a transient net magnetization on an ultrafast time scale. This phenomenon is attributed to the asymmetric charge accumulation and the OISTR between the two Ni magnetic sublattices, facilitated by the intrinsic structural and electronic asymmetry of the Janus architecture. Furthermore, we identify distinct spin dynamics across the layers: the Ni_1_ and Ni_2_ sites exhibit different demanganization governed by spin-polarized charge transfer processes. This asymmetric interlayer interaction effectively stabilizes the transient ferrimagnetic state. Our work elucidates the microscopic mechanisms of ultrafast magnetization dynamics in 2D CFiMs and demonstrates the potential of Janus structures such as NiICl for controlling nonequilibrium magnetic states, providing valuable insights for the design of ultrafast spintronic and opto-magnetic devices.

## Methods

To identify the spin dynamics of the NiICl bilayer under the influence of ultrafast laser pulses, a fully noncollinear spin-dependent version of real-time time-dependent density functional theory (rt-TDDFT) calculations was used. The time evolving state functions (ψ) were calculated by solving the time-dependent Kohn–Sham (KS) equation as follows

$$i\frac{\partial \psi_{j}\left(r,t\right)}{\partial t}=\left[\frac{1}{2}{\left(-i\nabla +\frac{1}{c}{Α}_{ext}\left(t\right)\right)}^{2}+\upsilon_{s}\left(r,t\right)+\frac{1}{2c}\sigma \cdot {Β}_{s}\left(r,t\right)+\frac{1}{4c^{2}}\sigma \cdot \left(\nabla \upsilon_{s}\left(r,t\right)\times \left(-i\nabla \right)\right)\right]\psi_{j}\left(r,t\right)$$

where **Α**_**ext**_(*t*) and σ represent vector potential and Pauli matrices. The KS effective potential υ_s_(**r**,*t*) = υ_ext_(**r**,*t*) + υ_H_(**r**,*t*) + υ_xc_(**r**,*t*) can be decomposed into external potential υ_ext_, classical Hartree potential υ_H_, and exchange-correlation (XC) potential υ_xc_, respectively. The KS magnetic field can be written as **Β**_**s**_(**r**,*t*) = **Β**_**ext**_(**r**,*t*) + **Β**_**xc**_(**r**,*t*), where **Β**_**ext**_ and **Β**_**xc**_ represent the magnetic field of the applied laser pulse with an additional magnetic field and an XC magnetic field, respectively. The last term in eq 1 stands for the spin–orbital coupling (SOC) effect. The magnetization density can be calculated as $m\left(r,t\right)=\underset{j}{\sum }\psi_{j}^{*}\left(r,t\right)\sigma \psi_{j}\left(r,t\right)$ and the integral of this vector field over the unit cell leads to the spin angular momentum.

Calculations at the ground state were performed at the density functional theory (DFT) level using the Vienna ab initio simulation package (VASP),^35,36^ using the Perdew–Burke–Ernzerhof (PBE) exchange-correlation functional.^37^ The Brillouin zone was represented by a Γ Monkhorst–Pack grid of 11 × 11 × 1 for structure relaxation and electronic structures of the NiICl bilayer. A correction of *U* = 3 eV only for the electronic structures of the Ni element is employed.^38^ An energy cutoff of 500 eV was used to determine the self-consistent charge density for the plane wave basis sets. The structures were fully optimized until the maximum force on atoms was lower than 0.01 eV/Å and the total energy variation was lower than 1.0 × 10^–6^ eV. A vacuum of approximately 20 Å was added in the direction perpendicular to the slab model.

For rt-TDDFT simulations, we considered only the spin-polarized electron dynamics based on the Born–Oppenheimer approximation. The electron–phonon coupling effects are not taken into account. Photoinduced dynamics calculations were made using a fully noncollinear version of rt-TDDFT and a full-potential augmented plane-wave ELK code.^39^ A regular mesh in a *k*-space of 8 × 8 × 1, a smearing width of 0.027 eV, and a time step of Δ*t* = 0.1 au were used to simulate excited dynamics. The laser pulses that were used in this study were linearly polarized at a selected frequency. All calculations were performed using adiabatic local spin density approximations (ALSDA)^40^ with the correction of *U* = 3 eV for Ni atoms.

## Supplementary Material



## Notes

(Figure S1) The charge density difference of the NiICl bilayer. (Figure S2) The band structures of the NiICl bilayer and the magnetic moments of the Ni atoms at different *U* values (PDF)
