# Supplementary material for: Ultrafast Spin Dynamics in 2D Fully Compensated Ferrimagnets: A Time-Dependent Ab Initio Study
Source: J Phys Chem Lett. 2025 Oct 17;16(43):11128–33. doi: 10.1021/acs.jpclett.5c02874 (PMC12581156; doi:10.1021/acs.jpclett.5c02874)
Supplement: Supplementary file 1 [file jz5c02874_si_001.pdf]

Supplementary Information

Ultrafast Spin Dynamics in 2D Fully Compensated Ferrimagnets: a Time-dependent Ab initio Study

Shuo Li [1*], Ran Wang [1], Thomas Frauenheim [1,2], Sandong Guo,[3] Zhaobo Zhou [4], Junjie He [1,4*]

1 Institute for Advanced Study, Chengdu University, Chengdu 610106, China

2 School of Science, Constructor University, Bremen, 28759, Germany

3 School of Electronic Engineering, Xi'an University of Posts and Telecommunications, Xi'an 710121, China

4 Department of Physical and Macromolecular Chemistry, Faculty of Science, Charles University, Prague 12843, Czech Republic

* Corresponding authors.

Email*: lishuo@cdu.edu.cn, junjie.he@natur.cuni.cz*

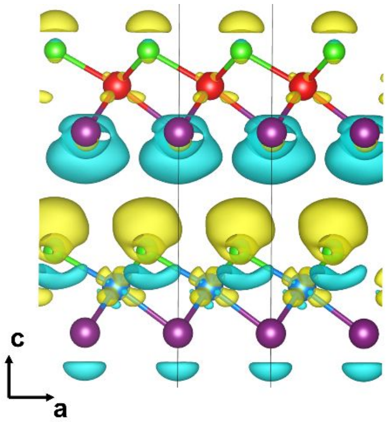

Figure S1 The charge density difference of the NiICl bilayer. Yellow and blue domains indicate the charge accumulation and dissipation, respectively. The isosurface is set to 0.00005 *e*/Bohr^3.

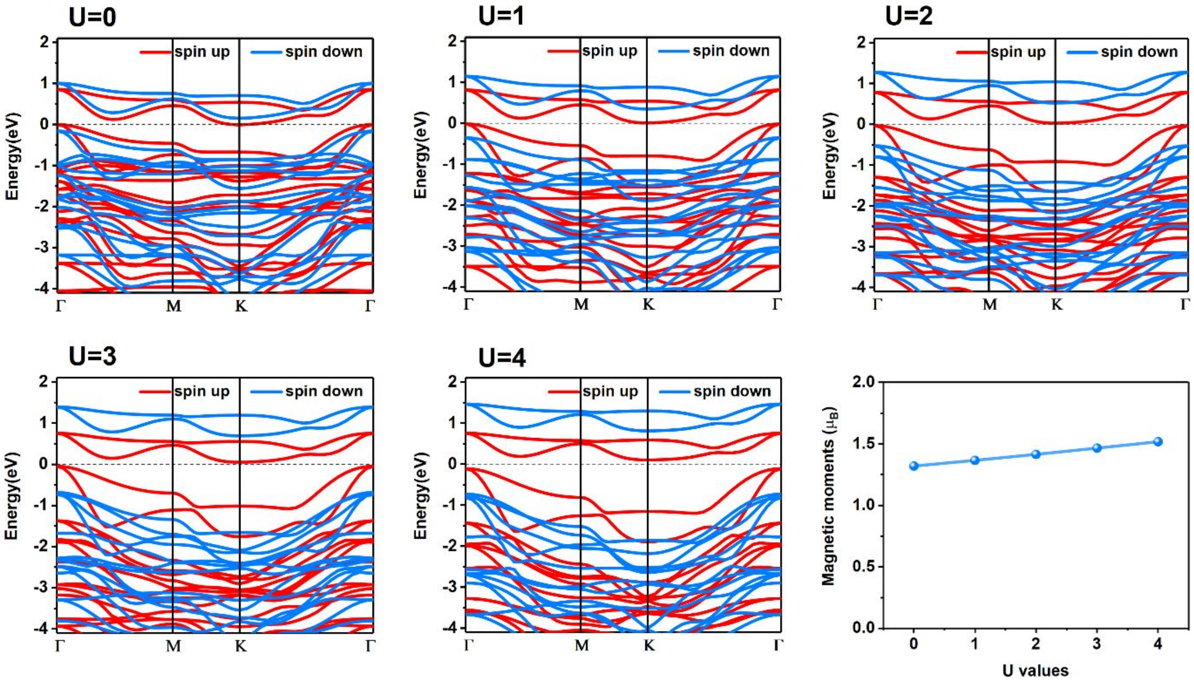

Figure S2 The band structures of NiICl bilayer and the magnetic moments of the Ni atoms at different U values.
